# Comparison of laparoscopic hepatectomy and radiofrequency ablation for small hepatocellular carcinoma patients: a SEER population-based propensity score matching study

**DOI:** 10.1007/s13304-024-02016-w

**Published:** 2024-10-01

**Authors:** Xi Wang, Xinqun Chai, Ruiya Tang, Yunjie Xu, Qinjunjie Chen

**Affiliations:** Department of Hepatobiliary Surgery, Union Hospital, Tongji Medical College, Huazhong University of Science and Technology, Wuhan, China

**Keywords:** Small hepatocellular carcinoma, Prognosis, Laparoscopic hepatectomy, Radiofrequency ablation, Propensity score matching

## Abstract

This study was designed to compare the efficacy of laparoscopic hepatectomy (LH) and radiofrequency ablation (RFA) in terms of their therapeutic effect on small hepatocellular carcinoma (SHCC). The SEER database was employed to integrate SHCC patients who had received treatment with either LH (n = 1132) or RFA (n = 797). The LH group (n = 623) and the RFA group (n = 623) were matched with 1:1 propensity score matching (PSM) in order to reduce the possibility of selection bias. The Kaplan–Meier method and Cox proportional hazards regression method were employed to ascertain the prognostic factors associated with overall survival (OS) and disease-specific survival (DSS). Both before and after PSM, the 1, 3 and 5-years OS and DSS were significantly higher in the LH groups compared to the RFA group. Besides, for SHCC with tumor size ≤ 2cm (n = 418), even P values not reaching statistical significance, the survival curves were compatible with a superiority of LH over RFA for OS and DSS in overall (P = 0.054 and P = 0.077), primary SHCC (P = 0.110 and P = 0.058) and recurrent SHCC (P = 0.068 and P = 1.000) cohorts. In contrast, for SHCC with tumor size between 2 and 3 cm (n = 828), LH group always had a better OS and DSS in the all cohorts (all P < 0.05). In addition, higher AFP level, poor differentiation grade, recurrent tumor and treatment type were independent prognostic factors for OS, while poor differentiation grade, larger tumor size and treatment type were the independent prognostic factors for DSS (all P < 0.05). LH was associated with better OS and DSS than RFA in SHCC patients. Even in tumor size ≤ 2 cm, LH still should be the first choice as its long-term survival benefits.

**Supplementary Information:**

The online version contains supplementary material available at 10.1007/s13304-024-02016-w.

## Introduction

Hepatocellular carcinoma (HCC) is one of the most common malignant tumors in the world, ranking sixth in incidence and third in mortality [1]. For small HCC (SHCC) (tumor diameter ≤ 3 cm), liver transplantation is the most effective treatment to achieve complete cure, especially for patients combined with cirrhosis [2–4]. However, liver transplantation has not been widely used in clinical practice due to various limitations such as major trauma, high cost, high incidence of postoperative complications and donor source [5, 6].

Radical hepatectomy is the preferred treatment for SHCC when patients' liver function and performance status are tolerable [7–9]. Compared with other therapies, radical hepatectomy can remove as much gross tumor tissue as possible while minimizing the occurrence of complications, offering a greater chance of cure and long-term survival [10–12]. With advances in laparoscopic techniques, laparoscopic hepatectomy (LH) has been widely used to treat resectable SHCC, which plays an important role in reducing postoperative recurrence and prolonging patient survival [13, 14]. Radiofrequency ablation (RFA) is often considered as an alternative treatment for SHCC patients who have impaired liver function, specific tumor location or other conditions that make radical hepatectomy intolerable [15–17]. Takayama et al. have shown that RFA and radical hepatectomy have comparable treatment outcomes and similar survival rates in SHCC patients, with less trauma and lower complication rates [18]. However, Kutlu et al. concluded that overall survival (OS) as well as disease-specific survival (DSS) was shorter in RFA than LH [19]. The major difference might be related to the presence of microvascular invasion (MVI) [20].

There has been controversy regarding the first-line treatment modality for patients with SHCC, and numerous studies have demonstrated that LH and RFA both provide a favorable prognosis for SHCC patients. Therefore, the aim of our study is to provide a basis for the optimal treatment choice for SHCC by comparing the long-term survival outcomes of SHCC patients treated with LH and RFA.

## Materials and methods

### Study population

We used SEER*Stat 8.3.2 software to extract clinical data from the SEER 17 registry database (2010–2020). The database collects cancer diagnosis and survival data on approximately 30% of the United States population. Patients with tumor size ≤ 3 cm who diagnosed as HCC with reliable pathological results (site code C22.0) from 2010 to 2019 were included. Patients with missing survival data or clinical data, with evidence of metastatic disease (including lymph node metastasis), with malignant tumor of other sites, with other treatment type (except for LH and RFA), and younger than 18 years or older than 95 years were excluded. Time-to-event was derived from the date of diagnosis or the date of terminal event (either death or last follow-up). The study was exempted from approval by the research ethics committee of our institution because the SEER data were de-identified and made publicly available for research use.

Data related to year of diagnosis, patient characteristics (age at diagnosis, gender and ethnicity), alpha-fetoprotein (AFP) level, tumor characteristics (pathological differentiation grade, AJCC stage, T stage, tumor size and whether it was recurrent or not), treatment type and survival follow-up (vital status, cause of death and months of survival) were included in the analysis. OS was defined as the interval between initial diagnosis and death from any cause. DSS was defined as the interval between initial diagnosis and death attributed to SHCC.

### Statistical analysis

SPSS 26.0 and R 4.3.1 software were used for data analysis. Continuous variables were expressed as mean ± standard deviation and compared by Student’s t test. Categorical variables were expressed as frequencies and percentages and compared by chi-squared test or Fisher's exact test. 1:1 propensity score matching (PSM) was used to reduce the possibility of selection bias between the LH and RFA groups. The caliper adopted for PSM was 0.02 and the variables included in PSM were age, sex, race, AFP, differentiation grade, AJCC stage, T stage, tumor size, and tumor recurrence. Univariate and multivariate Cox proportional hazards regression analysis was used to obtain the hazard ratio (HR) and 95% confidence interval (CI) for prognostic factors for OS and DSS. Then, OS and DSS were analyzed in both groups using the Kaplan–Meier method and differences between survival curves were assessed using the log-rank test. A two-tailed *P* value < 0.05 was considered the measure of statistical significance.

## Results

### Patient characteristics

**Fig. 1:**
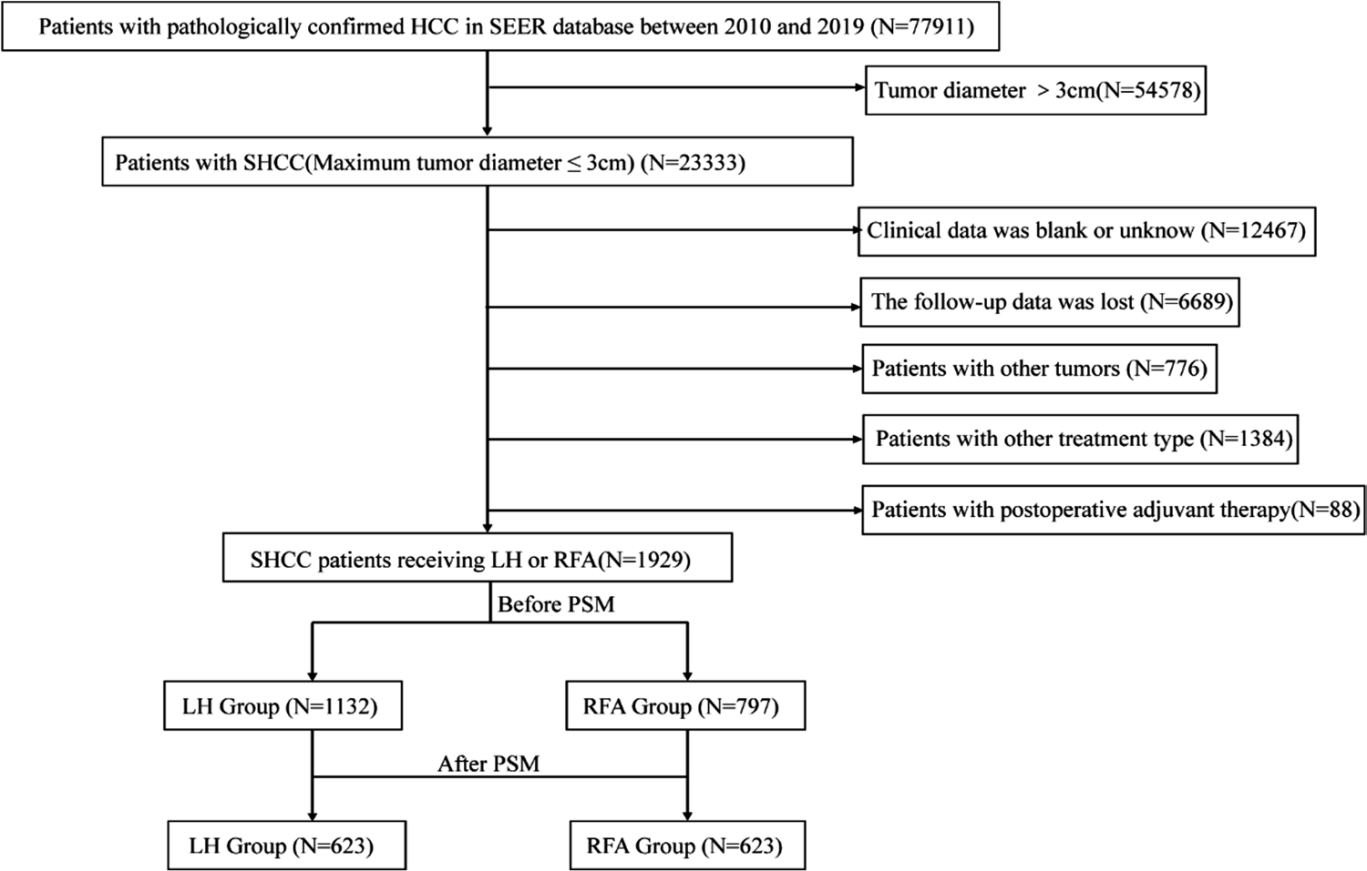
The flowchart of SHCC patients enrolled in this study

A total of 1929 SHCC patients receiving LH or RFA were included in this study, with a median follow-up time of 43.4 months (Fig. 1). There were significant differences in the following variables between LH and RFA group: race, AFP, differentiation grade and T stage (all *P* < 0.05). After 1:1 PSM, there was no significant difference between LH and RFA group, and the two groups were comparable (Table 1).

**Table 1:** Baseline characteristics of the study patients

Variables	Before matching				After matching			
	LH (n = 1132)	RFA (n = 797)	P value	SD value	LH (n = 623)	RFA (n = 623)	P value	SD value
Patient characteristics								
Age			0.887	0.007			0.216	0.070
< 60years	350 (30.9)	244 (30.6)			197 (31.6)	177 (28.4)		
≥ 60years	782 (69.1)	553 (69.4)			426(68.4)	446(71.6)		
Sex			0.582	0.025			0.216	0.068
Male	808(71.4)	578(72.5)			475 (76.2)	456(73.2)		
Female	324 (28.6)	219 (27.5)			148 (23.8)	167 (26.8)		
Race			< 0.001	0.195			0.674	0.004
White	626 (55.3)	562 (70.5)			427 (68.5)	437 (70.1)		
Asian	344 (30.4)	133 (16.7)			129 (20.7)	115 (18.5)		
Black	143 (12.6)	75 (9.4)			56 (9.0)	56 (9.0)		
Others	19 (1.7)	27 (3.4)			11 (1.8)	15 (2.4)		
AFP			0.003	0.055			0.489	0.066
Negative	405 (35.8)	288 (36.1)			231 (37.1)	215 (34.5)		
Borderline	217(19.2)	108(13.6)			99 (15.9)	94 (15.1)		
Positive	510 (45.1)	401 (50.3)			293 (47.0)	314 (50.4)		
Tumor characteristics								
Differentiation grade			< 0.001	0.198			0.500	0.029
I/II	918 (81.1)	708 (88.8)			567 (91.0)	560 (89.9)		
III/IV	214 (18.9)	89 (11.2)			56 (9.0)	63 (10.1)		
AJCC stage			0.072	0.078			0.946	0.010
I	857 (75.7)	571 (71.6)			485 (77.8)	486 (78.0)		
II	258 (22.8)	217 (27.2)			138 (22.2)	137 (22.0)		
III and above	17 (1.5)	9 (1.1)			–	–		
T stage			0.008	0.055			0.945	0.010
T1	859 (75.9)	577 (72.4)			487 (78.2)	488 (78.3)		
T2	258 (22.8)	217 (27.2)			136 (21.8)	135(21.7)		
T3 and above	15 (1.3)	3 (0.4)			–	–		
Tumor size (cm)			0.295	0.048			0.904	0.007
≤ 2	414 (36.6)	273 (34.3)			210 (33.7)	208 (33.4)		
2–3	718 (63.4)	524 (65.7)			413(66.3)	415 (66.6)		
Tumor recurrence			0.303	0.048			0.278	0.059
No	930 (82.2)	640 (80.3)			531 (85.2)	517 (83.0)		
Yes	202 (17.8)	157 (19.7)			92 (14.8)	106 (17.0)		

*PSM* propensity score matching, *LH* laparoscopic hepatectomy, *RFA* radiofrequency ablation, *AFP*, alpha fetoprotein, *SD* standardized differences

### Independent prognostic factors for SHCC

We applied combined univariate and multivariate Cox regression analysis to screen independent prognostic factors of OS and DSS in SHCC. Before PSM, the univariate analysis revealed that age, sex, AFP, AJCC stage, T stage, tumor recurrence and treatment type were significantly associated with OS and the multivariate analysis revealed that age, sex, AFP, AJCC stage, T stage, tumor recurrence and treatment type were independent prognostic factors of OS (all *P* < 0.05, Supplementary Table S1). Besides, the univariable results revealed that age, sex, AFP, differentiation grade, AJCC stage, T stage, tumor size and treatment type were significantly associated with DSS and the multivariate results revealed that age, sex, AFP, differentiation grade, AJCC stage, T stage, tumor size and treatment type were independent prognostic factors of DSS (all *P* < 0.05, Supplementary Table S2).

After 1:1 PSM, the univariate analysis revealed that age, AFP, differentiation grade, AJCC stage, T stage, tumor recurrence and treatment type were significantly associated with OS and the multivariate analysis showed that AFP, differentiation grade, tumor recurrence and treatment type were independent prognostic factors of OS (all *P* < 0.05). However, age, differentiation grade, AJCC stage, T stage, tumor size and treatment type were significantly associated with DSS and the multivariate analysis showed that differentiation grade, tumor size and treatment type were the independent prognostic factors of DSS (all *P* < 0.05, Table 2).

**Table 2:** Univariate and multivariate Cox regression analysis for OS and DSS after PSM

Variable	Univariate analysis				Multivariate analysis		
	HR	95%CI	P value	HR		95%CI	P value
OS							
Age (≥ 60years)	1.247	1.032–1.507	0.022	1.160		0.959–1.404	0.126
Sex (Male)	1.091	0.891–1.336	0.397				
Race							
White	Ref	Ref	Ref				
Asian	0.669	0.524–0.854	0.001				
Black	0.880	0.650–1.190	0.406				
Others	0.672	0.334–1.354	0.266				
AFP							
Negative	Ref	Ref	Ref	Ref		Ref	Ref
Borderline	1.535	1.194–1.972	< 0.001	1.625		1.263–2.091	< 0.001
Positive	1.228	1.008_1.496	0.042	1.217		0.996–1.488	0.055
Differentiation grade (III/IV)	1.476	1.127–1.931	0.005	1.485		1.131–1.951	0.004
AJCC stage (II)	1.308	1.074–1.593	0.008	0.486		0.068–3.467	0.471
T stage (T2)	1.329	1.091–1.619	0.005	2.843		0.396–20.403	0.299
Tumor size (2-3cm)	1.171	0.975–1.406	0.090				
Tumor recurrence (Yes)	1.355	1.087–1.689	0.007	1.413		1.130–1.767	0.002
Treatment type (LH)	0.535	0.446–0.642	< 0.001	0.537		0.447–0.645	< 0.001
DSS							
Age (≥ 60years)	1.284	1.021–1.615	0.033	1.209		0.960–1.523	0.107
Sex (Male)	1.180	0.920–1.514	0.193				
Race							
White							
Asian	0.669	0.497–0.901	0.008				
Black	1.023	0.725–1.442	0.899				
Others	0.867	0.409–1.836	0.709				
AFP							
Negative	Ref	Ref	Ref	Ref		Ref	Ref
Borderline	1.447	1.067–1.962	0.017				
Positive	1.201	0.949–1.521	0.128				
Differentiation grade (III/IV)	1.519	1.101–2.096	0.011	1.465		1.060–2.026	0.021
AJCC stage (II)	1.433	1.136–1.808	0.002	0.664		0.093–4.748	0.683
T stage (T2)	1.451	1.149–1.832	0.002	2.162		0.301–15.554	0.444
Tumor size (2-3cm)	1.338	1.068–1.677	0.011	1.299		1.035–1.631	0.024
Tumor recurrence (Yes)	1.122	0.845–1.489	0.427				
Treatment type (LH)	0.511	0.410–0.638	< 0.001	0.512		0.410–0.640	< 0.001

### Survival analysis

**Fig. 2:**
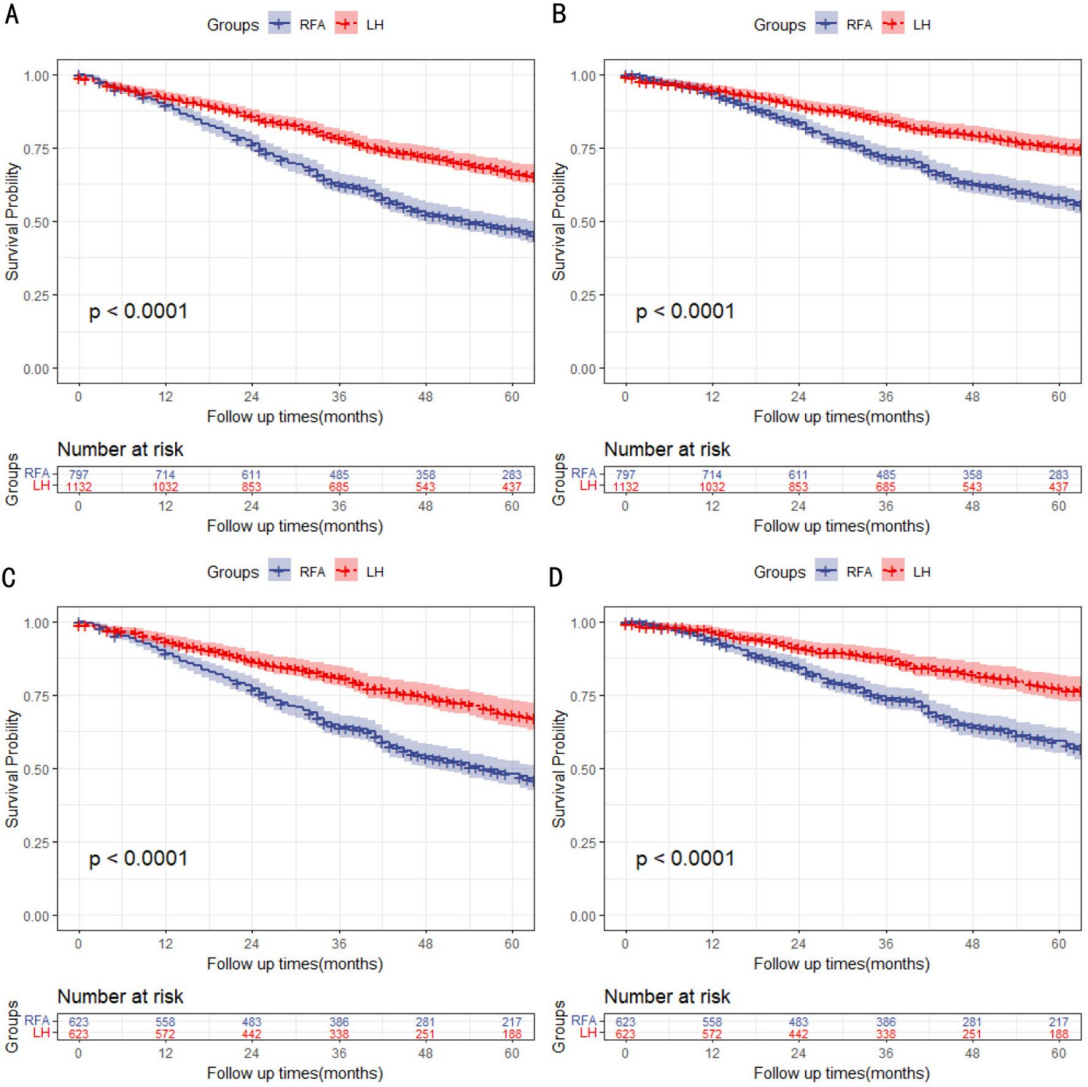
OS and DSS of LH group and RFA group in overall cohort. (**A** OS in overall cohort before PSM; **B** DSS in overall cohort before PSM; **C** OS in overall cohort after PSM; **D** DSS in overall cohort after PSM)

Compared with the RFA group, the LH group had better OS and DSS both before and after PSM (all *P* < 0.05, Fig. 2). Before PSM, the median follow-up periods were 43.5 months (range 21.1–65.9 months) and 43.1 months (range 21.0–65.2 months) in the LH and RFA group, with 389 patients (34.4%) in the LH group and 471 patients (59.1%) in the RFA group who died. The 1-year, 3 year and 5 year OS rates were 92.3%, 80.4% and 65.6% in the LH group and 90.2%, 63.0% and 40.9% in the RFA group (*P* < 0.001), respectively. The 1 year, 3 year, and 5 year DSS rates were 95.1%, 86.2% and 76.8% in the LH group, 93.7%, 59.6% and 57.5% in the RFA group (*P* < 0.001), respectively.

After PSM, the median follow-up periods were 40.1 months (range 18.3–61.9 months) and 43.3 months (range 21.4–65.2 months) in the LH and RFA group, with 183 patients (27.4%) in the LH group and 356 patients (57.1%) in the RFA group who died. The 1 year, 3 year and 5 year OS rates were 93.4%, 83.5% and 71.0% in the LH group and 89.2%, 64.5% and 42.9% in the RFA group (*P* < 0.001), respectively. The 1 year, 3 year, and 5 year DSS rates were 96.6%, 89.6% and 81.7% in the LH group, 94.0%, 75.9% and 60.0% in the RFA group (*P* < 0.001), respectively (Supplementary Table S3).

### Subgroup analysis based on primary or recurrent SHCC

**Fig. 3:**
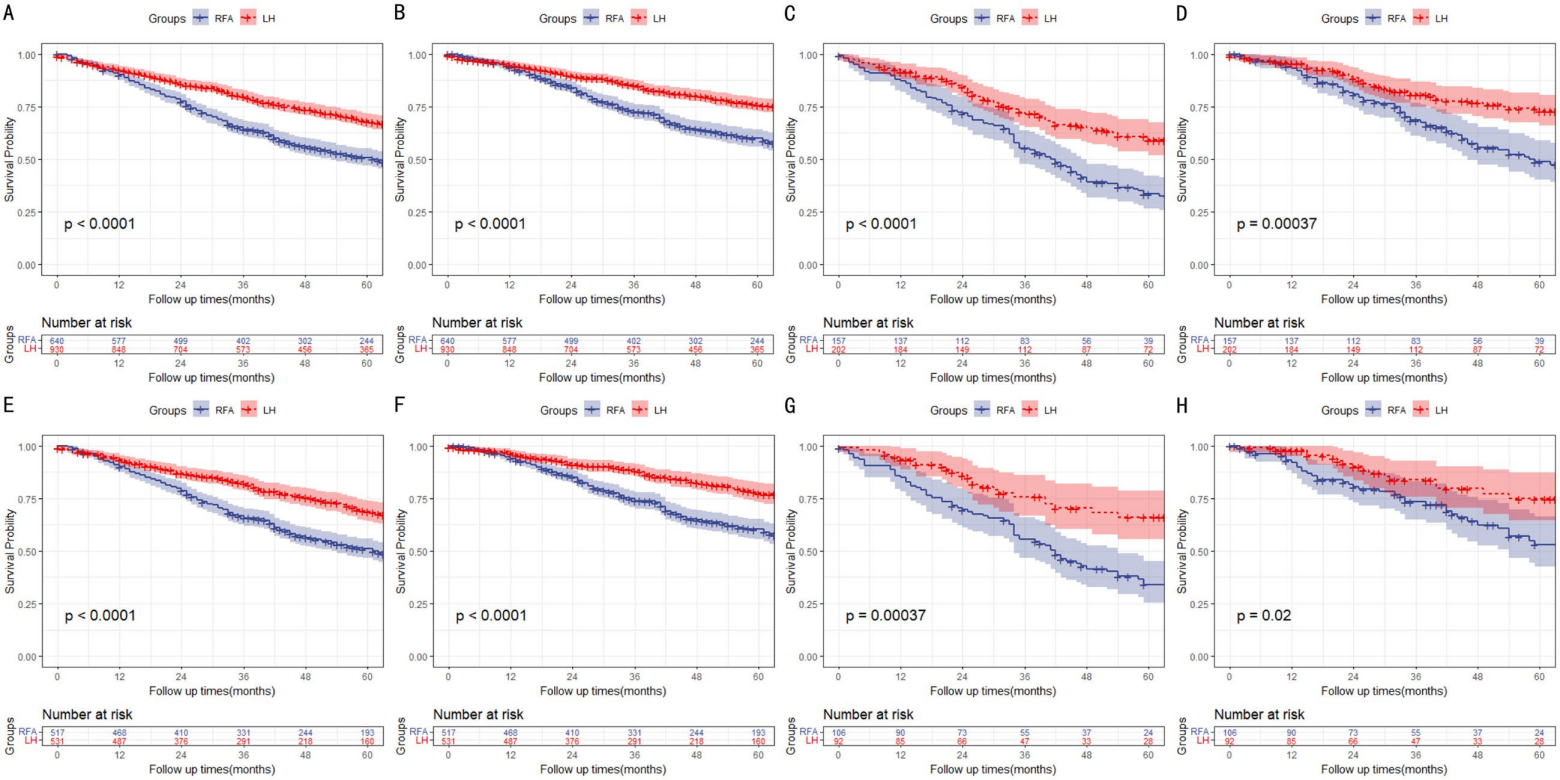
OS and DSS of LH group and RFA group in primary and recurrent SHCC cohort. (A, OS in primary SHCC cohort before PSM; B, DSS in primary SHCC cohort before PSM; C, OS in recurrent SHCC cohort before PSM; D, DSS in recurrent SHCC cohort before PSM; E, OS in primary SHCC cohort after PSM; F, DSS in primary SHCC cohort after PSM; G, OS in recurrent SHCC cohort after PSM; H, DSS in recurrent SHCC cohort after PSM)

All patients were divided into primary (n = 1570) and recurrent (n = 359) cohorts according to whether the SHCC was primary or not. In primary SHCC cohort, LH group had a better OS and DSS than RFA group before and after PSM (*P* < 0.001), respectively. In recurrent SHCC cohort, OS and DSS were also better in the LH group than RFA group before and after PSM (*P* < 0.001, Fig. 3), respectively.

### Subgroup analysis based on tumor size after PSM

**Fig. 4:**
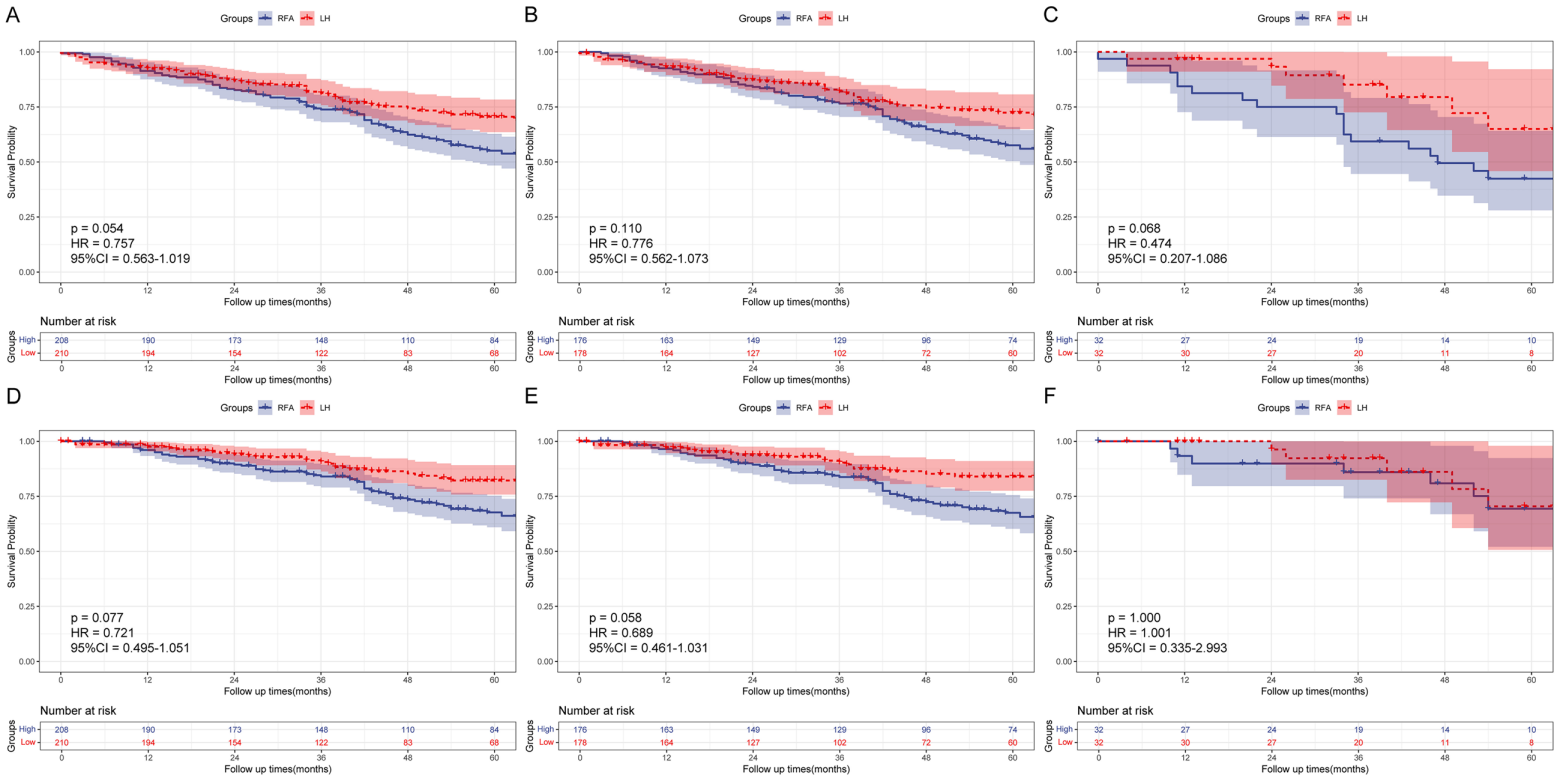
OS and DSS of LH group and RFA group for patients with tumor size ≤ 2 cm in overall cohort, primary SHCC cohort, and recurrent SHCC cohort after PSM. (A, OS in overall cohort; B, OS in primary SHCC cohort; C, OS in recurrent SHCC cohort; D, DSS in overall cohort; E, DSS in primary SHCC cohort; F, DSS in recurrent SHCC cohort)

**Fig. 5:**
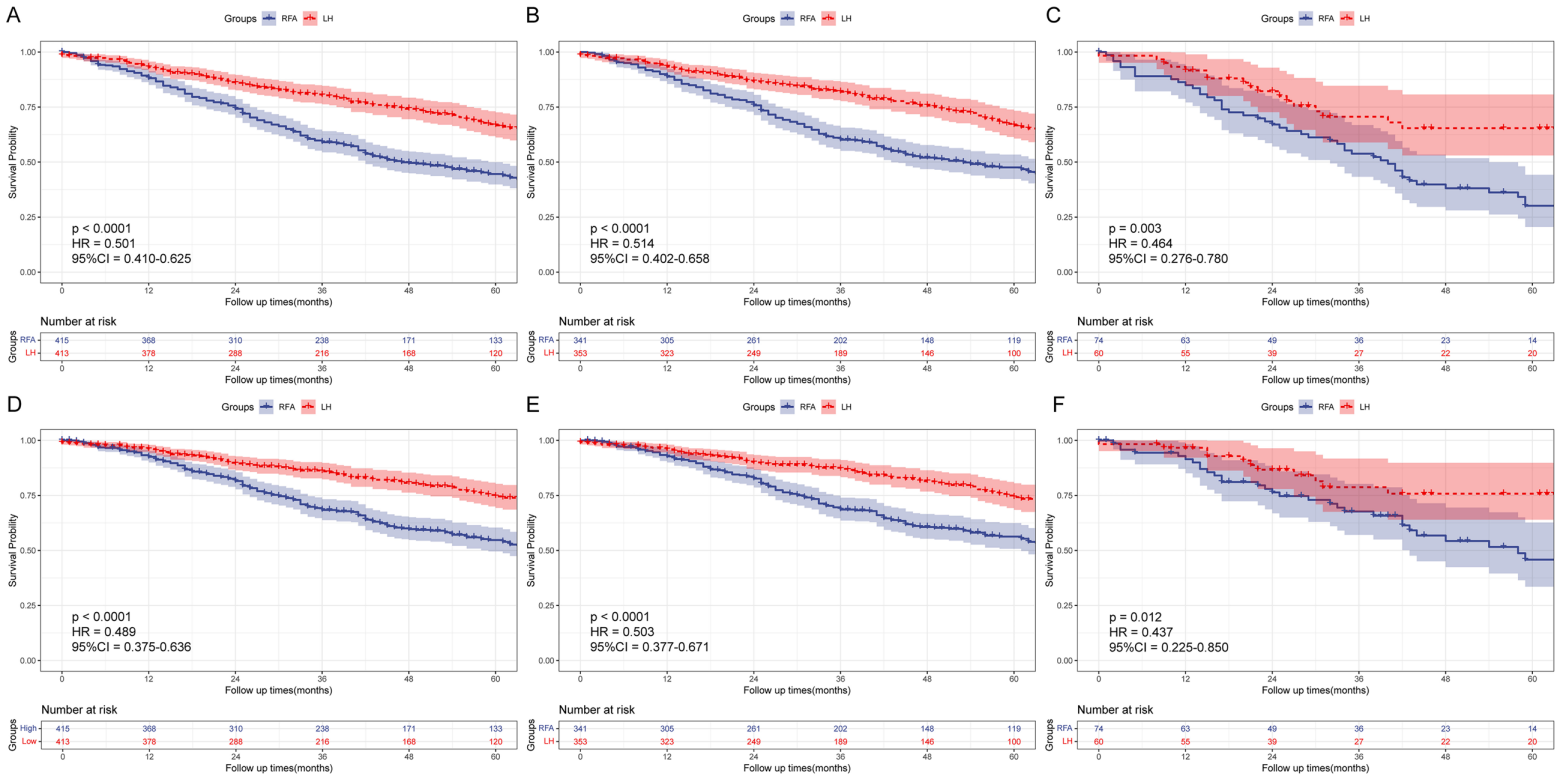
OS and DSS of LH group and RFA group for patients with tumor size between 2 and 3 cm in overall cohort, primary SHCC cohort, and recurrent SHCC cohort after PSM. (**A** OS in overall cohort; **B** OS in primary SHCC cohort; **C** OS in recurrent SHCC cohort; **D** DSS in overall cohort; **E** DSS in primary SHCC cohort; **F** DSS in recurrent SHCC cohort)

For SHCC with tumor size ≤ 2 cm (n = 418), analysis showed that there was no significant statistical difference between LH and RFA groups for OS (HR = 0.757, 95%CI: 0.563–1.019, *P* = 0.054) and DSS (HR = 0.721, 95% CI 0.495–1.051, *P* = 0.077). Furthermore, further stratification based on primary and recurrent factors revealed similar statistical outcomes. In primary cohort (n = 354), OS (HR = 0.776, 95% CI 0.562–1.073, *P* = 0.110) and DSS (HR = 0.689, 95% CI 0.461–1.031, *P* = 0.058) between LH and RFA groups were no significant statistical difference. In recurrent cohort (n = 64), there was also no significant difference between LH and RFA group for OS (HR = 0.474, 95%CI: 0.207–1.086, *P* = 0.068) and DSS (HR = 1.001, 95% CI 0.335–2.993, *P* = 1.000, Fig. 4). It was noteworthy to observe that despite the P values exceeding 0.05, the survival curves were compatible with a superiority of LH over RFA in SHCC with tumor size ≤ 2 cm. In contrast, for SHCC with tumor size between 2 and 3 cm (n = 828), LH group always had a better OS and DSS in the overall, primary SHCC and recurrent SHCC cohorts (all *P* < 0.05, Fig. 5).

## Discussion

The choice of surgical approach is extremely important in the treatment of SHCC. According to the American Association for the Study of Liver Diseases practice guidelines, LH should be the standard treatment for HCC [21]. However, for SHCC patients who are not suitable for LH, RFA can be used as an alternative treatment to improve patient survival [22, 23]. With the continuous advancement of medical technology, more and more studies have confirmed that RFA has similar OS as LH [18, 24, 25], however, there is still some controversy about the efficacy of RFA in the treatment of SHCC. The aim of our study was to compare the therapeutic effect of LH and RFA in SHCC patients. After using PSM to reduce patient selection bias, our study showed that in total, LH had better OS and DSS than RFA. However, when tumor ≤ 2 cm, RFA had comparable efficacy to LH and could be used as an alternative treatment to LH.

In our study, LH was superior to RFA in terms of OS and DSS in overall SHCC patients, which is consistent with previous studies. Shin et al. reported that LH was superior to RFA in terms of OS, showing better oncological outcomes [26]. Another meta-analysis also found that in SHCC patients, LH provided better long-term survival outcomes at 1, 3 and 5-years OS compared with RFA [27]. Although most studies have shown that RFA has inferior DSS and OS to LH, only a few studies have found similar long-term survival outcomes between RFA and LH [28–30]. Most researchers point out that RFA has a higher postoperative early recurrence [31, 32]. The biggest advantage of LH is that LH could resect the primary tumor, MVI and satellite foci as much as possible. However, it is undeniable that RFA has some advantages in terms of minimally invasiveness, which is associated with fewer postoperative complications, shorter operative time and shorter hospital stay [33, 34].

Previous studies have shown that, under certain specific conditions, LH and RFA have similar outcomes in OS and DSS. For primary SHCC with tumor size ≤ 2 cm, LH and liver transplantation is the first choice, but for patients with impaired liver function and no indication for liver transplantation, RFA may be the first-line option [20]. Mironov et al. found that there was no significant difference between LH and RFA in terms of OS or DSS [35]. Huang et al. reported similar results for LH and RFA in terms of long-term survival outcomes in primary SHCC with tumor size ≤ 2 cm [36]. Besides, numerous studies have shown that for recurrent SHCC with tumor size ≤ 2 cm, LH provides better local disease control and reduces early recurrence, but long-term survival of LH and RFA is similar [37–40]. However, in our study, for recurrent SHCC with tumor size ≤ 2 cm, LH and RFA had similar OS and DSS, which contradicted previous results. The reason might be that we included fewer recurrent SHCC patients.

In our study, survival analysis showed an interesting result that the P values exceeded 0.05 between the LH and RFA groups in the comparison of OS and DSS for patients with tumor size ≤ 2 cm, which seems no significant prognosis difference. However, the survival curves showed that although in the initial postoperative period, RFA showed similar survival benefits to LH for patients with tumor size ≤ 2 cm, for the long-term survival outcomes, the survival curves were consistent with a superiority of LH over RFA. Therefore, we believe that, despite P values did not reach the statistical significance, LH should be the more favorable approach to improve prognosis, as previously elucidated, due to its advantage in tumor clearance.

As medical technology continues to evolve, the notion of a multi-parameter treatment hierarchy has emerged as a pivotal topic of contemporary discourse in the realm of HCC. A growing consensus among researchers posited that the management of HCC should prioritize survival benefits, transcending the traditional focus solely on tumor staging [41]. This perspective was echoed in another seminal work, which underscored the selection of treatment methods must be guided by outcomes, encompassing the patients' frailty level, complication, tumor location, biological characteristics, progression patterns, liver function, technological constraints, and resource availability, rather than relying solely on tumor staging [42]. Our research endeavored have fortified this viewpoint, further validating the paramount importance of adopting a multi-parameter therapeutic approach in the management of HCC. Upon meticulous analysis, we recommend that for both primary and recurrent SHCC with tumor size ≤ 2 cm, RFA might be served as an effective means to minimize intraoperative morbidity and facilitate favorable short-term prognosis. However, LH emerged as the optimal strategy for achieving more favorable long-term survival outcomes. Therefore, in the context of clinical decision-making, the choice of surgical approach should be tailored to the specific circumstances and patient characteristics, ensuring an individualized and evidence-based approach to care.

In addition, we found that higher AFP level, poor differentiation grade, recurrent tumor and treatment type (RFA vs. LH) were independent prognostic factors of OS, besides, poor differentiation grade, larger tumor size and treatment type (RFA vs. LH) were independent prognostic factors of DSS, which is consistent with previous studies [43–46].

Undeniably, our study encountered some limitations. Firstly, inherent to its retrospective design, the SEER database was prone to biases, including selection and information biases, which could potentially compromise the accuracy and reliability of our research findings. Secondly, the database's constrained variable availability, notably the absence of critical laboratory parameters and treatment modalities, restricts the comprehensiveness and precision of our results, hindering a deeper exploration of disease mechanisms and the development of targeted therapeutic strategies. In addition, Des-γ-carboxy-prothrombin (DCP) has been shown to play an important role in the prognosis of HCC [47, 48], but the SEER database did not have information on this and therefore was not included in the study. Finally, there were insufficient data on whether patients underwent adjuvant therapy after surgery, which might also have an impact on the results.

## Conclusion

In conclusion, LH demonstrated superior efficacy over RFA in the treatment of SHCC. Moreover, for tumor size ≤ 2 cm, even both therapeutic modalities yielded comparable survival benefits in the short-term, LH should still be the first choice because of its better long-term outcomes. Our study highlights the need for individualized treatment strategies that take into account not only tumor size but also patient-specific factors and disease characteristics.

## Supplementary information

Below is the link to the electronic supplementary material.

Supplementary file1 (DOCX 27 KB)

## Data Availability

Availability of data and materials Data from SEER database are available in the Surveillance, Epidemiology, and End Results cancer registry (https://seer.cancer.gov) and can also be provided by the corresponding author.
