# Supplementary material for: Comparison of laparoscopic hepatectomy and radiofrequency ablation for small hepatocellular carcinoma patients: a SEER population-based propensity score matching study
Source: Updates Surg. 2024 Oct 1;76(8):2755–66. doi: 10.1007/s13304-024-02016-w (PMC11628577; doi:10.1007/s13304-024-02016-w)
Supplement: Supplementary file 1 — Supplementary file1 (DOCX 27 KB) [file 13304_2024_2016_MOESM1_ESM.docx]

Supplementary Table S1. Univariate and multivariate Cox regression analysis in OS before PSM

| Variable | Univariate analysis | | | Multivariate analysis | | |
| --- | --- | --- | --- | --- | --- | --- |
|  | HR | 95%CI | P value | HR | 95%CI | P value |
| Age (≥ 60years) | 1.292 | 1.113-1.500 | 0.001 | 1.257 | 1.081-1.461 | 0.003 |
| Sex (Male) | 1.206 | 1.033-1.407 | 0.017 | 1.225 | 1.049-1.430 | 0.011 |
| Race |  |  |  |  |  |  |
| White | Ref. | Ref. | Ref. |  |  |  |
| Asian | 0.558 | 0.467-0.667 | 0.001 |  |  |  |
| Black | 0.933 | 0.756-1.153 | 0.523 |  |  |  |
| Others | 0.694 | 0.422-1.140 | 0.149 |  |  |  |
| AFP |  |  |  |  |  |  |
| Negative | Ref. | Ref. | Ref. | Ref. | Ref. | Ref. |
| Borderline | 1.452 | 1.197-1.760 | <0.001 | 1.578 | 1.300-1.915 | <0.001 |
| Positive | 1.284 | 1.101-1.497 | 0.001 | 1.299 | 1.113-1.517 | 0.001 |
| Differentiation grade (III/IV) | 1.185 | 0.992-1.416 | 0.061 |  |  |  |
| AJCC stage |  |  |  |  |  |  |
| I | Ref. | Ref. | Ref. | Ref. | Ref. | Ref. |
| II | 1.384 | 1.193-1.606 | <0.001 | 1.355 | 1.167-1.573 | <0.001 |
| III and above | 2.825 | 1.744-4.576 | <0.001 | 0.998 | 0.372-2.678 | 0.996 |
| T stage |  |  |  |  |  |  |
| T1 | Ref. | Ref. | Ref. | Ref. | Ref. | Ref. |
| T2 | 1.382 | 1.191-1.604 | <0.001 | 1.355 | 1.167-1.573 | <0.001 |
| T3 and above | 4.527 | 2.611-7.849 | <0.001 | 6.314 | 2.039-19.550 | 0.001 |
| Tumor size (2-3cm) | 1.126 | 0.978-1.297 | 0.098 |  |  |  |
| Tumor recurrence (Yes) | 1.374 | 1.169-1.614 | <0.001 | 1.388 | 1.177-1.636 | <0.001 |
| Treatment type (LH) | 0.572 | 0.500-0.655 | <0.001 | 0.571 | 0.498-0.654 | <0.001 |

Supplementary Table S2. Univariate and multivariate Cox regression analysis in DSS before PSM

| Variable | Univariate analysis | | | Multivariate analysis | | |
| --- | --- | --- | --- | --- | --- | --- |
|  | HR | 95%CI | P value | HR | 95%CI | P value |
| Age (≥60years) | 1.324 | 1.106-1.584 | 0.002 | 1.307 | 1.091-1.565 | 0.004 |
| Sex (Male) | 1.240 | 1.030-1.493 | 0.023 | 1.260 | 1.045-1.518 | 0.015 |
| Race |  |  |  |  |  |  |
| White | Ref. | Ref. | Ref. |  |  |  |
| Asian | 0.544 | 0.439-0.675 | <0.001 |  |  |  |
| Black | 0.954 | 0.742-1.225 | 0.710 |  |  |  |
| Others | 0.798 | 0.459-1.386 | 0.423 |  |  |  |
| AFP |  |  |  |  |  |  |
| Negative | Ref. | Ref. | Ref. | Ref. | Ref. | Ref. |
| Borderline | 1.473 | 1.167-1.859 | <0.001 | 1.618 | 1.280-2.045 | <0.001 |
| Positive | 1.350 | 1.123-1.622 | <0.001 | 1.285 | 1.066-1.548 | 0.009 |
| Differentiation grade (III/IV) | 1.306 | 1.063-1.605 | 0.011 | 1.365 | 1.103-1.688 | 0.004 |
| AJCC stage |  |  |  |  |  |  |
| I | Ref. | Ref. | Ref. | Ref. | Ref. | Ref. |
| II | 1.549 | 1.301-1.844 | <0.001 | 1.467 | 1.230-1.749 | <0.001 |
| III and above | 3.963 | 2.404-6.535 | <0.001 | 1.537 | 0.572-4.130 | 0.394 |
| T stage |  |  |  |  |  |  |
| T1 | Ref. | Ref. | Ref. | Ref. | Ref. | Ref. |
| T2 | 1.542 | 1.295-1.835 | <0.001 | 1.467 | 1.230-1.749 | <0.001 |
| T3 and above | 6.234 | 3.507-11.081 | <0.001 | 5.195 | 1.653-16.330 | 0.005 |
| Tumor size (2-3cm) | 1.244 | 0.978-1.297 | 0.012 | 1.241 | 1.044-1.474 | 0.014 |
| Tumor recurrence (Yes) | 1.200 | 0.982-1.467 | 0.075 |  |  |  |
| Treatment type (LH) | 0.540 | 0.459-0.634 | <0.001 | 0.518 | 0.439-0.612 | <0.001 |

Supplementary Table S3. The outcome and HR of LH and RFA groups for SHCC.

| Parameter |  | Before PSM | | After PSM | |
| --- | --- | --- | --- | --- | --- |
|  |  | LH | RFA | LH | RFA |
| OS |  |  |  |  |  |
|  | 1-year | 92.3% | 90.2% | 93.4% | 89.2% |
|  | 3-year | 80.4% | 63.0% | 83.5% | 64.5% |
|  | 5-year | 65.6% | 40.9% | 71.0% | 42.9% |
|  | HR (95%CI) | 0.576 (0.504-0.659) | | 0.531 (0.442-0.637) | |
|  | P value | < 0.001 | | < 0.001 | |
| DSS |  |  |  |  |  |
|  | 1-year | 95.1% | 93.7% | 96.6% | 94.0% |
|  | 3-year | 86.2% | 59.6% | 89.6% | 75.9% |
|  | 5-year | 76.8% | 57.5% | 81.7% | 60.0% |
|  | HR (95%CI) | 0.542 (0.461-0.636) | | 0.508 (0.407-0.634) | |
|  | P value | < 0.001 | | < 0.001 | |
